# How has sustainable development goals declaration influenced health financing reforms for universal health coverage at the country level? A scoping review of literature

**DOI:** 10.1186/s12992-021-00703-6

**Published:** 2021-04-23

**Authors:** Walter Denis Odoch, Flavia Senkubuge, Charles Hongoro

**Affiliations:** 1 African Centre for Health Systems Development (ACHSD), Plot 2703, Block 208 Bombo rd., P.O Box 21743, Kampala, Uganda; 2 East Central and Southern Africa Health Community, Plot 157 Njiro Rd Arusha, P.O Box 1009, Arusha, Tanzania; 3 School of Health Systems and Public Health, Faculty of Health Sciences, University of Pretoria, Gauteng Province, 0028 South Africa; 4 Development, Capable and Ethical State (DCE) Division, Human Sciences Research Council of South Africa, Private Bag X41, Pretoria, 0001 South Africa

**Keywords:** Health financing reform, Universal health coverage, Sustainable development goals, Review of literature

## Abstract

**Background:**

Achieving universal health coverage (UHC) requires health financing reforms (HFR) in many of the countries. HFR are inherently political. The sustainable development goals (SDG) declaration provides a global political commitment context that can influence HFR for UHC at national level. However, how the declaration has influenced HFR discourse at the national level and how ministries of health and other stakeholders are using the declaration to influence reforms towards UHC have not been explored. This review was conducted to provide information and lessons on how SDG declaration can influence health financing reforms for UHC based on countries experiences.

**Methods:**

We conducted a rapid review of literature and followed the preferred reporting items for systematic review and meta-analysis (PRISMA) guideline. We conducted a comprehensive electronic search on Ovid Medline, PubMed, EBSCO, Scopus, Web of Science. In searching the electronic databases, we combined various conceptual terms for “sustainable development goals” and “health financing” using Boolean operators. In addition, we conducted manual searched using google scholar.

**Results:**

Twelve articles satisfied our eligibility criteria. The included articles were analyzed thematically, and the results presented narratively. The SDG declaration has provided an enabling environment for putting in place necessary legislations, reforming health financing organization, and revisions of national health polices to align to the country’s commitment on UHC. However, there is limited information on the process; how health ministries and other stakeholders have used SDG declaration to advocate, lobby, and engage various constituencies to support HFR for UHC.

**Conclusion:**

The SDG declaration can be a catalyst for health financing reform, providing reference for necessary legislations and policies for financing UHC. However, to facilitate better cross-country learning on how SDG declaration catalyzes HFR for UHC there, is need to examine the processes of how stakeholders have used the declaration as window of opportunity to accelerate reforms.

**Supplementary Information:**

The online version contains supplementary material available at 10.1186/s12992-021-00703-6.

## Background

Global declarations shape public policy priorities and guide development finance flows [1–3]. The declarations energize governmental processes and provide a reference point that guides national policies toward priority development issues. Therefore, global and regional declarations can be powerful tools for shaping policy and programme response on issues that are of public concern [4]. The United Nations General Assembly (UNGA) meeting in New York in September 2015 under the theme, *“Transforming our world: the 2030 Agenda for Sustainable Development”,* adopted 17 sustainable development goals (SDG) as the post millennium development goals (MDGs) development blue print [5]. Health is explicitly addressed in paragraph 26 of the SDGs declaration [6]:

*To promote physical and mental health and well-being, and to extend life expectancy for all, we must achieve universal health coverage and access to quality health care. No one must be left behind...*

Achieving universal health coverage (UHC) is one of the overarching targets of the 2030 agenda for sustainable development, under goal 3 *(Ensure healthy lives and promote well-being for all at all ages)*. Universal health coverage is based on the principle that all people should have access to health services they need and do not suffer financial hardship while accessing the services [7]. This implies an effective, efficient and equitable health financing system is a critical and essential component that contributes to achievement of UHC target under the SDG declaration [8–11]. It is only when resources are adequate, efficiently used and equitably mobilized, pooled and spent that all people can enjoy sustained progress towards UHC [12, 13]. Health financing influences progress on the three UHC goals of equity in the use of health services, quality of care and financial protection through effects on UHC intermediary objectives of transparency and accountability, efficiency and equity in resource distribution [8, 14].

For many countries, achieving UHC require reforms in their health financing systems so that people have financial protection while accessing quality health care [15]. Therefore, a number of countries in the African region have been attempting to reform the way health is financed to ensure sustainable progress towards UHC [15–19]. However, these attempts, largely spearheaded by Ministries of Health have been slow and intermittent as reflected in the slow and/or forward and backward movements in the reform processes [16, 20–22]. The process of introducing comprehensive National Health Insurance Scheme (NHIS), for example, has been ongoing in the last 2 to 3 decades in Uganda, Kenya, Zambia and South Africa [16, 20–24]. In South Africa, Uganda, Kenya and Zambia, Health Insurance Bills were (or have been) drafted, however they have not reached parliament for legislation. In cases where the parliament passed the Act, it never became Law due to failure by the Executive arm of governments to ascent to the Bills or the process took very long [22, 25, 26]. However, the policy landscape where various efforts for strengthening or reforming health financing systems occur may be different with the SDG declaration. This is because health financing reforms are a political process, and SDG is a global political declaration with UHC as one of its targets. Effective, efficient and equitable health financing systems are critical for the achievement of UHC [27, 28]. Therefore, given the overarching importance of UHC as an SDG target, it is likely that the declaration has influenced health financing reforms and if so, lessons on the how and the outcomes of those reforms are relevant for intercountry learning. So far, there has been no comprehensive review of literature on how health financing reform processes have been influenced by the SDG declaration at national level. Therefore, through this study we aimed at contributing towards filling this knowledge gap and more broadly to the 2013 WHO [29] call for evidence based research that inform initiatives to advance UHC.

## Methods

### Study design, review questions and definition

We registered the protocol a priori in PROSPERO (CRD42020194090) because initially we thought we could conduct a systemic review. However, due to resource constraints, we ended up conducting a scoping review of literature. We thus present here an overview of the methodological approach and highlight minor differences between the protocol and the actual conduct of the review. We conducted a review of findings from studies and reports on how the SDG declaration is influencing or has influenced health financing reforms for UHC. For this review, health financing reforms for UHC is conceptualized to refer to changes in arrangement and management of health financing system sub-functions of revenue collection, pooling, and purchasing, and policy on benefits design and rationing towards efficient and equitable system [30]. The reforms explored are linked or can be attributed to have resulted from global discourses on sustainable development agenda or SDG declaration.

In order to improve the transparency and methodological robustness of this scoping review of litertaure, we followed preferred reporting items for systematic review and meta-analysis (PRISMA) guidelines [31], where we defined the review questions; identified, selected and appraised the studies; abstracted the data; and synthesized and interpreted the results. The review questions were: a) How has the SDG declaration influenced the process of health financing reform for UHC? b) What dimensions of health financing have been influenced by the SDG declaration? and c) How have Ministries of Health and other stakeholders used the SDG declaration to influence health financing reforms for universal health coverage?

### Criteria for considering studies for the review

All study designs were considered for the review. This was to account for the complex nature of health financing reforms. Specifically, we included randomized and non-randomized studies, evaluation studies, policy analyses, stakeholder analyses, and peer-reviewed case studies and commentaries. Proposals and studies published in abstracts only were excluded.

The inclusion criteria based on population, intervention (exposure) and outcome (PEO) [32], required that the study reviewed: *(i) Is* on health systems reform, or other government reforms where health financing is a part of the reform process, or is specifically on health financing reforms, and is published between Jan 2012 and June 2020. This date was chosen to include studies conducted after the 2012 Rio de Janeiro United Nations Conference on Sustainable Development, where the process of developing SDG was initiated. (ii) Describes how SDG declaration has influenced reforms in health systems financing for UHC as the intervention. (iii) Reports on changes to at least one of the following health financing dimensions of management or organization i.e., revenue collection, pooling, purchasing, and policy on benefits design and rationing as the outcome.

### Search methods for identification of studies

A comprehensive electronic search of six databases was conducted using indexed and free text words in the following databases: Ovid Medline, PubMed, EBSCO, Scopus, Web of Science between March and September 2020 by WDO and FS. In searching the electronic databases, various conceptual terms for “sustainable development goals” and “health financing” were searched. We used the Boolean operators ‘OR’ to combine the terms within each concept and ‘AND’ to combine the two concepts. We did not use search filter for study type, language (we anticipated to use the google translate for non-English articles), country or geographical area in order to find as many studies on the topic as possible. In addition, we screened the reference lists of included studies from the databases for additional eligible studies. We also conducted manual search for relevant articles from publications on websites of WHO, online journals (health systems and reforms, health policy and planning), UHC2030 partnership, and used the search engine google scholar.

In order to gauge the viability of the review, we first conducted a pilot electronic search on PubMed using the aforementioned terms. The search yielded 692 potential articles. From initial screening of titles and abstracts, 69 articles were considered potentially eligible. Of the 69 articles, one was in Chinese and one in Spanish, the rest were in English. From the 69 articles, we were able to retrieve 68 full length articles, the exception being the one published in Chinese. The article in Spanish was translated using google scholar. After review of full length articles of the 68 studies, two studies were found to meet our eligibility criteria; Agustina et al. [33] and Wang and Zhou [34]. In addition to gauging viability of the review, the pilot search enabled improvement of our screening and eligibility, data abstraction and quality assessment tools in terms of appropriateness and uniform application of criteria across reviewers (WDO and FS), thus enhancing the validity of the process and minimizing bias in the study identification and selection.

### Data collection, extraction and analysis

#### Selecting studies

**Fig. 1:**
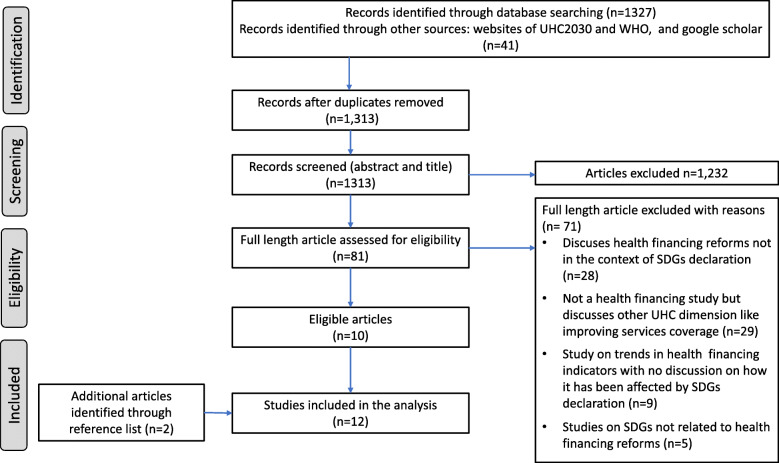
PRISMA flow chart

All retrieved articles from the databases were exported to EndNote X9 [35], where duplicates were removed. The titles and abstracts of identified articles were screened for potential eligibility. Full text of studies judged as potentially eligible were retrieved. The text retrieved were screened in detail for eligibility, using a standardized screening form (Additional file 1: Appendix 1) in duplicate. The number of studies included and excluded are documented and illustrated in the PRISMA flow diagram (Fig. 1). The full texts of all relevant studies found to meet the inclusion criteria were retained for the final synthesis [36]. The studies that failed to meet the inclusion criteria at the full-text screening phase were documented with reasons for the exclusion from the analysis.

#### Data extraction

Data was abstracted using standardized data abstraction form adapted from the Joanna Briggs Institute (JBI) data abstraction format (Additional file 1: Appendix 2) [37]. Summary of data extraction (Table 1) has been provided so that readers can assess the critical review process for this study. The study characteristics extracted included the bibliographic details of study (study title, author, year of publication), objectives (purpose of the study), study design, setting (country); influence of SDGs declaration on health financing reforms; the dimension or aspect of health financing system reformed; and how ministries of health and other stakeholders have used SDGs declaration to drive health financing reform for UHC (Table 1).

**Table 1:** Characteristics of the included study and findings

Author, Year, Country, study design	How has SDGs influenced the process of health financing reforms (HFR) for UHC?	What dimensions of health financing have been influenced by SDGs declaration?	How stakeholders used the declaration to influence HFR for UHC
Agustina et al [33], 2019, Indonesia, *Study design:* Qualitative, documentary review	– SDGs led to discussions on health financing sustainability for UHC – Establishment of a scheme that was adaptable, accommodate diverse needs, assures financial risk protection – In 3 years (by 2019), the NHIS became the largest single-payer health insurance scheme in the world	*Revenue Collection* – Intense solicitation of payments from self-enrolled members – Categorization of contributors/sources of revenue for pooled fund *Pooling of funds* – Reduced fragmentation in risks pools – Establishment of Social Security Agency for Health (SSAH) – Law of mandatory % allocation of government budget to health *Purchasing* – A Single payer for UHC established – Payment-capitation and diagnostic-related group based on Indonesia tariff. *Policy on benefit entitlements* – Scope of services covered by capitation payment is determined by Indonesian Medical Council – Decrease in fee-for-service payment	
Fahim et al [38], 2018, Bangladesh, *Study design: Mixed method, documentary review*	– The national health policy 2011–2032 updated to address contemporary issues of SDG and UHC – Health financing policy emphasizing solidarity in financing, equity of access and provision of quality care	*Pooling of funds* – Policy emphasis on allocating a significant percent of government spending to health – Exploring ways of reducing OOP	
Chilufya and Kamanga [39], 2018, Zambia, *Study design:* Commentary *Study aim:*	– Zambia’s transformational health agenda is in tandem with SDGs target 3.8 – Health sector strategic plan 2017–2021 was informed by the SDG agenda – Country building on the progress past health reforms during SDG era.	*Revenue Collection* – Exploring ways of implementing sustainable health care financing – Mandatory pre-payment contribution being established *Pooling of funds* – Commitment to allocating sufficient government funding for health – Establishment of NHIF fund as a pooling agency *Purchasing* – Exploring reforming payment mechanism from inactive to active purchasing	
Ahmadnezhad et al. [40], 2019, Iran, *Study Design:* Quantitative, document review	– Health transformation plan recalibrated to form part of government commitment on SDG agenda	*Purchasing* – Ministry of Health and Medical Education (MOHME) reduced co-payment *Policy on benefit entitlements* – Basic health insurance coverage extended from 83.2% of population to 93.2% – Aim to decrease prevalence of catastrophic expenditure to less than 1% by end of 2021	
Lee et al. [41], 2019, Republic of Korea, *Study design:* Qualitative, documentary review	– In 2017, government announced NHI reform ‘Moon Jae-in care’ to increase coverage rate to 70% by 2022 and its considered a government’s commitment to health-related SDG – Reinforcing the benefits and financial coverage of national health insurance (NHI) is a core aspect of the reform – The advent of SDGs and inclusion of the President’s name in the health financing reform indicates how seriously the government has taken health financing reforms for UHC	*Policy on benefit entitlements* – The population already covered, reform focuses on increasing the scope/depth of coverage and reducing cost-sharing – Reducing out-of-pocket (OOP) from the cost sharing component to minimize the catastrophic and impoverishing expenditure	
Nagpal et al. [42], 2019, LPDR, *Study design:* Quantitative, review of surveys data	– Approved national health insurance (NHI) Law in 2017 – To achieve the health targets in the SDG and meet new and emerging challenges, the Government of LPDR accelerated its efforts towards universal health coverage, e.g. the nationwide scale-up of free at point of care MCH services – The NHI was quickly rolled out in 15 provinces by the end of 2017, and covered the entire country except Vientiane capital by the end of 2018	*Pooling of funds* – In 2016/17, free MCH program was consolidated with 3 other social protection schemes into a single national health insurance scheme (reducing number of risk pools), *Purchasing* – User fee payment by pregnant women and children under 5 replaced by case-based payment under the MCH initiative	
Capuno et al. [43], 2018, Philippines, *Study design:* Mixed method, depth interviews and documentary review	– Duterte government’s aims to attain the health-related SDG targets through extending health insurance coverage to all, thus ensuring each Filipino “financial freedom when accessing services”, – The Philippines Health Agenda 2016–2022 was informed by and has taken into consideration Philippines commitment to SDG agenda – Main health goals and strategic policies including on health financing are reflected in the Mid-term Philippine Development Plan developed based on ambition to achieve SDG targets	*Revenue Collection* – Continued preservation of Sin Tax for health – Earmarking has helped to sustain progress towards achieving of SDG target on UHC *Purchasing* – PhilHealth as single purchasing agency *Policy on benefit entitlements* – Poor, marginalized and vulnerable protected from cost of health care through Sin Tax	– There is stronger link between DOH’s national objective for health and the national development plan following the SDG declaration – DOH was successful in generating political and financial support to pursue universal health access and in legislating various proposal e.g. Sin Tax Law
Dayrit et al. [44], 2018, Philippines, *Study design*: Health system review, mix method	– SDGs has informed Philippine Health Agenda [Administrative Order No. 2016–0038] which is about reforming PhilHealth into main national purchaser of health services – SDGs is seen as facilitator of natural progression towards universal health access	*Pooling of funds* – Changes in pooling arrangement with mandatory PhilHealth cover *Purchasing* – Reformed capitation and no-balance billing arrangement for members – Fee-for-service phased out and case-rate payment applied by PhilHealth *Policy on benefit entitlements* – Increased benefit ratio -expanding enrollment of the poor in the NHIP & promoting quality of services	
Ranabhat et al. [45], 2019, Nepal, *Study design*: Systematic review	– National health system has prioritized achievement of UHC in line with UN SDG declaration	*Policy on benefit entitlements* – Free maternal and child health (MCH) services at point of care – Development of MCH service package – Nationwide scale-up of the scheme following limited geographic scope	– Lobby by visionary health care professionals, international organization and interest groups that consistently made reference to government commitment to SDGs led to establishment of national health insurance program. – In 2016 the government through Ministry of Health and Population started social health insurance scheme in some district and extended to 22 other districts by 2018.
Gera et al. [46], 2018, India, *Study design*: Commentary	– Government has taken important policy level initiatives in the recent years, especially after the launch of SDGs that include establishment of National Institution for Transforming India (NITI Aayog) and roll out of national health policy 2017. – The Integration of SDG agenda in NHP-2017 and NITI Aayog’s Vision for Health (2032) has provided an unprecedented opportunity for health financing reforms for UHC	*Pooling of funds* – Establishment of new flagship National Health Protection Scheme recently launched by the union government *Policy on benefit entitlements* – Exploration on how to incrementally expand coverage to cover larger population proportion and the range of services covered	– Leadership of federal Ministry of Health (MOH) has fostered a collaborative effort with other Government ministries and agencies, and state governments in the SDG era leading to the formation of national health protection scheme. – Effective stewardship from the federal MOH, reorganization of health care service delivery and strengtehing community participation and accountability.
Wang et al. [34], 2020, China, *Study design*: Qualitative, documentary review	– As part of Healthy China 2030, health is considered crucial entry-point to achieving SDGs because of its ability to lift people out of poverty – “Healthy China 2030” will improve access to essential health services covered by health insurance and financial assistance scheme	*Policy on benefit entitlements* – 95% of the population covered by health insurance schemes – Improved medical care insurance for targeted poverty-stricken population	
Tan et al [47], 2018, China, Study design: Commentary	– Healthy China 2030 was a response to the 2030 United Nations SDGs – A momentous endeavor to enhance public health	*Revenue Collection* – Encourage development of commercial health insurance schemes to supplement National Health Insurance Schemes *Policy on benefit entitlements* – Improved health insurance system targeting economically backward region – Healthy China 2030 has led to financial protection for the poor	

#### Appraisal of studies

We had planned to assess the quality of included articles for analysis using the Joanna Briggs Institute’s critical appraisal checklists for qualitative, quantitative and mixed-methods studies, however after reflections of the eligible articles, this was not conducted. This is because we included some articles that do not typically fall under conventional studies approaches such as commentaries and health system reviews, yet they met our eligibility criteria. Quality assessment was meant to support judgement on the relative contribution of each study to the development of explanations and relationships between SDG declaration and health financing reforms. However, given our review questions, quality assessment of articles would not have swayed these explanations of the relationship between SDG declaration and health financing reforms for UHC.

#### Synthesis and interpretation of results

We used NVIVo and a thematic framework (Additional file 1: Appendix 3) to facilitate analysis. Thematic framework synthesis is a qualitative approach that involves selecting, recording and categorizing key issues and themes [48]. For each article, the process involved familiarization with information, identification, recording, categorization and interpretation of the influence of SDG declaration on health financing reforms, and how Ministries of Health and other stakeholders have used the SDG declaration to advance health financing reforms towards UHC. We used Kutzin’s framework for analyzing health financing systems [8], to examine the changes in the organization and/or management of health financing functions. We then examined whether or not these changes were as result of the SDG declaration. Findings on the three review questions are presented narratively in the following section.

## Results

### Overview

The study selection process is summarized in the PRISMA flow chart (Fig. 1), while Additional file 1: Appendix 2 indicates the databases searched, searched dates and the yield. Out of 1313 citations, we identified 12 eligible studies. We excluded 71 articles at the full-text screening phase due to the following reasons;- the study did not discuss or indicate SDG declaration as a factor in health financing reform process, not a health financing study but discusses other UHC dimension like improving services coverage, study on trends in health financing indicators with no discussion on how it has been affected by SDG declaration, and studies on SDG not related to health financing reforms (see Additional file 1: Appendix 4, for reason of exclusion of each of the 71 articles).

### Characteristics of included studies and methodological appraisal

Table 1 describes the characteristics of the included studies in terms of study design, country of study, year of publication and summary of findings related to the three review questions. The twelve (12) articles included for analyses related the global sustainable development agenda or SDG declaration to health financing reforms, either as part of the background statements, findings, discussion or conclusion sections and made reference to a particular country. Two articles each were from China [34, 47], and Philippines [43, 44], and one each from Indonesia [33], India [46], Bangladesh [38], Zambia [39], Iran [40], Republic of Korea [41], Lao People’s Democratic Republic (LPDR) [42] and Nepal [45].

Three (3) of the articles can be described as mixed method studies, however they had limited or no statistical analysis rather simple trends or graphs of health financing quantitative indicators such as the level of out-of-pocket expenditure, health expenditure as proportion of total government expenditure and level of household impoverishment due to health spending [38, 43, 44]. Two studies were quantitative [40, 42], four were qualitative studies [33, 34, 41, 45], while three were commentaries [39, 46, 47]. The three commentaries were informed by personal experience and backed up by literature citations. Of the three mixed studies, one was a health system review using non-conventional approach to the conduct of research studies [44]. All studies and commentaries reviewed were based on data collected through documentary review except the one study by Capuno et al. [43], that also involved key informant interviews.

The focus of this study was on finding descriptions or explanations of the relationship between SDG declaration and health financing reforms for UHC. Therefore, we did not focus on the analyses of the quantitative aspect of the articles reviewed, rather we identified qualitative data from either the background, findings, and discussion and conclusion sections that provides explanation or describes the relationship between SDG declaration and HFR for UHC.

### Influence and use of SDG declaration in health financing reforms for UHC at national level

#### How has the SDG declaration influenced reforms in health financing for UHC?

On this review question, three themes emerged, these are: - national discussion on best ways to achieve UHC (agenda setting for UHC), legislation on health financing and budgets, and update or revision of national health (financing) policies and plans.

##### National discussion on how best to achieve UHC in the SDG era (agenda setting)

From the reviewed articles, the SDG declaration has informed, caused or intensified national level discussions of health financing for UHC [33, 34, 43–45, 47] and in some instances accelerated the pace with which governments considered the issue of UHC and its financing [41, 42, 46]. Agustina and colleagues [33], report that national level discussions in Indonesia on UHC scheme concluded that the scheme to be established should be adaptable, accommodative to the diverse needs and affordable as mandated by SDG declaration. These discussions resulted in consensus on four policy areas for implementation to ensure health financing sustainability for UHC, these were; − increase in premium to be paid by national health insurance contributing members, designing and implementation of cost containment measures, improving reimbursement process and promoting efficiency [33]. In Philippines, the Health Agenda 2016–2022 which include reforming PhilHealth as the single purchaser of health services and deepening health insurance coverage was informed by government’s commitment on SDG [43, 44]. While in Nepal, following the SDG declaration, the issue of financial risk protection when accessing health care has been prioritized by the government [45].

Tan and colleagues [47], reports that Healthy China 2030, a Chinse government blueprint on health (including financing of health care services especially for the poor regions) and socio-economic development was in response to SDG declaration. Health is considered a crucial entry point to achieving sustainable development goals, majorly due to its ability to lift people out of poverty [34]. In the Republic of Korea, the issue of improving financial coverage for the underprivileged has been on-going for years, however with the advent of SDG, this has now been taken more seriously by the government [41]. Nagpal et al. [42]*,* indicates that to achieve health targets in the SDG, the government of Lao People Democratic Republic (LPDR) accelerated its efforts toward UHC. While Gera and colleagues [46], report that the rollout of India’s national health policy had been delayed many times. However, this process was accelerated, demonstrating proactiveness by the government following the SDG declaration.

##### Legislation on health financing and budgets

The global discussions on sustainable development and the eventual SDG declaration in 2015 has led to the passing of specific Laws on health financing in some countries. As part of national reforms for achieving UHC, the Indonesian government passed a Health Law mandating that 5% of national budget goes to the health sector and local governments were directed to allocate 10% of its budget to health services [33]. In the LPDR, a national health insurance (NHI) Law was approved in 2017 as part of the national health financing strategy 2017–2020, which was informed to a great extent by the SDG declaration and the need to achieve UHC [42]. Following the global sustainable development declaration, the Philippine Government through an Administrative Order reformed the PhilHealth into the single and main national health purchasing agency with the aim of attaining health related SDG targets through extending health insurance coverage to all Filipinos [43, 44].

##### Revision of national health policies and plans

Reviewed studies indicate that countries updated their national health (especially financing) policies to address issues in the SDG [38, 39, 43, 46]. Bangladesh for example, updated its national health policy 2012–2032 to address contemporary issues of SDG and UHC [38]. In particular, the health financing policy objectives were revised to ensure reduction in out-of-pocket expenditure on health care to below 32% of total health expenditure [38]. In Zambia, the health sector strategic plan (HSSP) 2017 in which health related SDG targets are anchored focusses on six pillars, with reforming health financing to ensure UHC one of the key pillars [39]. In the Philippines, the health goals and strategic policies including on health financing have been developed based on the ambition to achieve SDG target on UHC [43]. India’s Government has taken important policy decisions after the launch of the SDG such as the establishment of National Institute for Transforming India and rolled out the national health policy (NHP) 2017 [46]. Gera et al. [46], reports that the SDG agenda was incorporated into NHP 2017 before it was rolled out and that this provides an opportunity for health financing reforms for UHC. China launched the Healthy China 2030 following the SDG declaration [47]. One of the major aim of Healthy China 2030 is the protection of citizens especially in poor region from financial risks associated with access to health care through reforming rural health insurance schemes [47].

#### What dimensions of health financing have been influenced by the SDG declaration?

The reviewed articles demonstrate that since the SDG declaration, there have been observable changes in either the process or outcome of the health financing functions, and policies that define and ratio benefit entitlements. These are elaborated below.

##### Revenue collection

Reviewed studies report changes in aspects of revenue collection or that countries are exploring alternative approaches to revenue collection for health services. Following the SDG declaration, Indonesian government through its Ministry of Health (MOH) embarked on the categorization of contribution into three sources of pooled funds, this was to enable identification of population proportion that has to be paid for through general tax revenue [33]. In addition, there has been intense solicitation of payments from self-enrolled members to national health insurance scheme [33]. In Zambia, the government has embarked on development of approaches for implementing a sustainable health care financing, especially the establishment of pre-payment contribution to supplement tax and donor funding [39]. Capuno and colleagues [43], report that the SDG declaration has made it easier the justification for continued allocation of 100% of Sin Tax to health in the Philippines. While in China, as part of Healthy China 2030, the government has encouraged the development of commercial health insurance schemes to supplement the national scheme [47].

##### Risk pooling and purchasing

Following global discussions and the declaration on sustainable development, the reviewed studies indicate that countries in effort to improve financing towards UHC established pooling agencies or transformed the operations of the existing national insurance pooling or purchasing agencies. In Indonesia, LPDR and Philippines, there have been consolidation and minimizing fragmentation of risk pools to improve efficiency [33, 42, 44]. Indonesia established Social Security Agency for Health (SSAH) as the main (single) national purchaser of health services [33] and the Philippines transformed PhilHealth into a single purchasing agency [43, 44]. In Bangladesh, the policy has emphasized solidarity in health financing and increased government allocation to health [38]. In Zambia the process of establishing a national health insurance fund as a pooling agency is in advanced stages [39]. Meanwhile in India a new national health protection scheme was established to provide health insurance cover by pooling risks, not covered by state governments [46].

On the purchasing, the payment mechanisms were changed from fee-for-service to capitation and/or diagnostic related group (DRG) based on the case-based payment in Indonesia, Philippines and LPDR [33, 42, 44]. Zambia’s government is also exploring ways of making Ministry of Health an active (strategic) purchaser as opposed to the current inactive purchaser of health services, including instituting a purchaser-provider split mechanism [39]. In Iran and Philippines, balance billing or co-payment were removed to drive down out-of-pocket payments [40, 44].

##### Policies that define and ration benefit

The reforms occasioned by SDG declaration on health financing has led to improvement in depth of coverage (extent to which covered services are covered) in Indonesia and Korea [33, 41], and increase in population coverage (proportion of population eligible for covered services) in Iran, Philippines, India and China [34, 40, 44, 46, 47]. The ‘Moon Jae-in’ reform launched in 2017 increased coverage rate by 70%, and reinforced benefits and financial protection provided by the national health insurance scheme in the Republic of Korea [41]. While in Iran, the basic health insurance coverage has been extended to 93.2% of the population [40]. The Philippines government has focused on extending coverage to the poor, marginalized and vulnerable population, ensuring they are protected from cost of health care through allocation of Sin Tax to cover for their premium [43]. In China by 2019, 95% of the population were covered by health insurance schemes thus increasing access with protection from financial risk [34]. In addition, the range of services covered under insurance schemes have been improved in the poverty-stricken regions of China [34, 47].

#### How ministries of health and other stakeholders are using SDG declaration to influence health financing reforms for universal health coverage?

Reviewed articles indicate that Ministries of Health have put efforts on fostering collaboration with other government agencies [46] and focused on linking health objectives with overall national development plans [43]. Capuno and colleagues reports that the Department of Health (DOH) established a strong link between national objective for health and the national development plan and national budget formulation process following the SDG declaration [43]. This was through generating successful political and financial support from the entire government. This has enabled the Ministry to pursue universal health care access agenda, including support in legalizing various proposal such as Sin Tax Law, where 100% goes into insurance coverage premium for the poor. In India, the leadership of national Ministry of Health and Welfare has actively fostered a collaborative effort with state governments leading to formation of national health protection scheme, to supplement state governments’ health insurance schemes. In addition, effective stewardship from national Ministry of Health and Welfare has led to reorganization of health care services delivery to implement a system-wide change that ensure financial protection against health costs through community participation and by being accountable [46]. Health care professionals and non-governmental organizations lobbied for the establishment of a national health insurance scheme in Nepal, arguing that health insurance will contribute to socio economic growth that will spur the achievement of other SDG targets [45].

## Discussion

In this review we explored how global discussions on sustainable development agenda and the SDG declaration influenced health financing reform processes for UHC at the national level. The review indicates that SDG declaration has influenced health financing reforms. The influence is majorly through two ways; (i) putting and sustaining health financing reforms (HFR) for UHC on the national agenda, and (ii) providing enabling context for debating and passing of Health Laws and reviewing of national health policies to strengthen health financing systems for UHC. Since the start of global discussions on sustainable development in 2012 and the eventual declaration on SDG in 2015, the issue of how to sustainably finance health more efficiently and equitably has dominated national discussions or gained momentum [33, 34, 41–47]. Countries have enacted Health Laws [33, 42], or reviewed and revised national health policies and strategies so that they are in tandem with the international commitment on SDG and UHC [38, 39, 43, 46]. These findings demonstrate the potential of SDG in promoting a more expansive and interrogative approach to creating systems for better health financing. This is through pushing various stakeholders to engage and seek ways of collaborating and finding appropriate mix of legislation, regulation, policies and guidelines to drive sustainable financing for UHC.

The outcome of the SDG declaration influence can be observed in changes (or proposed changes) to the organization and arrangement in health financing functions and policies. Countries such as Korea and China, where population coverage is over 90% are focusing on two of the three dimensions of UHC i.e., increasing the number of services covered and the extent to which a service is covered (depth of financial coverage), and not merely proportion of population coverage with basic health care. China and Korea are examples of countries demonstrating that UHC in journey, even when majority of population is covered with basic health care, breadth and depth of coverage can still be improved. The SDG declaration has provided a catalyst for the needed improvements [8, 49].

In terms of revenue collection, countries such as China, Republic of Korea and Indonesia are looking at covering the poor and indigent via general tax revenue. In addition, China is encouraging growth of commercial insurance schemes to supplement public health insurance scheme. Philippines is using Sin Tax (earmarked) to cover the premium for the poor while Indonesia is utilizing a Law that compels government to allocate a specific percentage to health sector budget. Zambia wants to develop national health insurance scheme as a way to supplement government general tax revenue and donor funding. In all these maneuvers by countries to improve available funding for health, the attainment of UHC as one of the SDG targets is being cited. However, as has been noted by Kutzin and WHO [8, 14, 50], it is prudent for governments to appreciate that all the revenues come from the people, in one way or another. Therefore, the focus should be on equity and efficient pooling and use of these resources.

Increasing the share of total public spending devoted to health, or increasing the level of compulsory prepaid revenues for health, strategic purchasing, minimizing fragmentation of risk pools, and simplification and promotion of the benefit package to increase people’s awareness of their benefits are pointers of reforms in health financing directed towards UHC [8]. Most of the reviewed articles point to the fact that countries’ changes to health financing functions are towards UHC. However, aspects such as encouraging commercial health insurance, as in China or explicit introduction of insurance scheme as a supplement to government and donor funding, as indicated for Zambia’s case may run counter to UHC aspiration and needs to be undertaken with caution.

Ministries of health and stakeholders within the health sector have leveraged on the SDG declaration to pursue health reforms through lobbying [45], and strategic engagement of stakeholders to generate political and financial support and buy-in into the reforms [43, 46]. The successful use by Ministries of Health and other stakeholders of government commitments to international agenda seems to depend in part on the leadership capacity and the ability to demonstrate that the health objectives are in tandem with overall national development plan and global aspirations. As Agyepong [51] argues, strong leadership and administrative capacity is required within countries to determine, design and implement contextually appropriate policies for UHC.

Given the political nature of reforms, approaches that make a political leader recognized or appear to be behind the reform such as including their name in the reform is another strategy that may work in some contexts, for a whole of government buy-in. This can be seen in the case with Republic of Korea and in the Philippines [41, 43, 44]. Active engagement of sub-national governments and other stakeholders by Ministries of Health can smoothen the process of introducing new health financing policies, as the case of India demonstrates [46]. The UHC2030 partnership [52] argues, “parliamentarians, ministers and local government officials play a major role [in] promoting, financing and implementing UHC, and their commitments and actions are critical for global commitments to be translated into local solutions”.

### Study limitation and research gaps requiring further studies

To the best of our knowledge this is the first review literature examining the relationship between SDG declaration and health financing reforms for UHC. The strength of our methodology includes the pre-publication of a protocol, a rigorous and transparent review process, and adherence to standard methods of reporting reviews. We searched multiple databases and screened references of identified articles.

We anticipated a limitation related to finding adequate number of studies reporting on reforms in health financing in countries that have been driven by SDG declaration. The reform processes take time and yet the SDG declaration was only made towards the end of 2015.. We addressed this by not restricting search by country, language or study design, and searching gray literature. This still remains a weakness in this study as we managed to get only 12 articles that were eligible (Fig. 1). However, this review remains a valuable addition to the field of health policy and systems research as it is the first review of literature exploring the linkage between SDG declaration and health financing reforms for UHC at the national level.

Only two of the 12 articles [38, 46], made attempts at describing how ministries of health and other stakeholders have utilized SDG declaration to influence health financing reforms for UHC. They identified lobbying, aligning health objectives to national development plan and budgeting process, and engagement of local governments while making reference to government commitments to SDG as some of the approaches used to influence health financing reform process. Apart from one study [43], all the reviewed articles used documentary review as sources of data. To fully understand how SDG declaration is influencing health financing reform processes for UHC, it is better to interview stakeholders at the national level. This is because document review only, is unlikely to unearth salient approaches being used by stakeholders in ensuring SDG declaration provides opportunity for health financing reforms for UHC. Through document reviews only, it is also difficult to examine other contextual factors including values that are influencing the use of SDGs declaration in reforms towards UHC. Therefore, it is difficult to draw conclusive lessons that can be adapted by stakeholders attempting to achieve similar outcomes reported in some of the reviewed articles as being influenced by the SDG declaration.

## Conclusion

Global declarations can be used as tools for lobbying, negotiation, re-strategizing and political mobilization. The SDG declaration has influenced reforms in health financing towards UHC by putting the issue on the national agenda, providing reference for the passing of national Laws to facilitate better health financing, and causing countries to revise their national health financing policies. The effects of these are observable in the processes or outcomes in health financing functions and polices including seeking alternative approaches to revenue collection and earmarking of revenue for health, improving pooling arrangements such as reduced fragmentations, pursuance of strategic purchasing methods, expanding coverage with a focus of all the three dimensions of UHC; population coverage, financial coverage and number of services paid through pool funds.

A critical gap in literature is the documentation of how the stakeholders, including Ministries of Health have used the declaration as opportunity to cause change in health financing towards UHC. Most authors only report on outcomes, while neglecting, what explains what happened; an aspect that are better appreciated through getting the perspectives of stakeholders through interviews. There is need for studies to document the countries experiences for shared learning on how the stakeholders in the health sector including Ministries of Health are utilizing global declaration; the SDG to foster desired reforms from their perspectives.

## Supplementary Information

**Additional file 1: Appendix 1.** Eligibility screening of articles. **Appendix 2**. Data abstraction form. **Appendix 3**. Thematic framework analysis format for summarizing changes in health financing occasioned by SDGs declaration. **Appendix 4**. Excluded studies and reasons for exclusion.

## Data Availability

The datasets used during the current study are available from the corresponding author on reasonable request.
