# Supplementary material for: How has sustainable development goals declaration influenced health financing reforms for universal health coverage at the country level? A scoping review of literature
Source: Global Health. 2021 Apr 23;17:50. doi: 10.1186/s12992-021-00703-6 (PMC8066969; doi:10.1186/s12992-021-00703-6)
Supplement: Supplementary file 1 — Additional file 1: Appendix 1. Eligibility screening of articles. Appendix 2. Data abstraction form. Appendix 3. Thematic framework analysis format for summarizing changes in health financing occasioned by SDGs declaration. Appendix 4. Excluded studies and reasons for exclusion. [file 12992_2021_703_MOESM1_ESM.docx]

**Appendix 1. Eligibility screening of articles**

***a) Screening for potential eligibility***

- Duplicate removal
- Title and abstract information screen against the following
  - Mention of health financing or health reforms in title or abstract
  - Mention on SDGs title or abstract
  - Published in 2012 or after

***b) Eligibility for inclusion of full-length articles for analysis***

*Abstract number:*

*Year of publication*

| ***Variables for assessments*** | ***Yes/No*** |
| --- | --- |
| Population (*health financing reforms, health systems reform with aspect on financing*) |  |
| Exposure (SDGs used to influence reforms: put on national agenda, the speed of the reforms, stakeholders-number, interest, power) |  |
| Outcomes (Description of the change in financing) |  |
| Impact (Equity, Protection, Quality) |  |
| Include |  |

**Results from the search strategy**

| Database | Years searched restricted to | Date Searched | Hits | Potentially eligible (Title/abstract) | Eligible |
| --- | --- | --- | --- | --- | --- |
| PubMed | 2000-2020 | 5 March 2020 | 692 | 42 | 2 |
| EBSCO* | 2000-2020 | 3 May 2020 | 126 | 44 |  |
| Ovid Medline | 2000-2020 | 2 July 2020 | 291 | 25 |  |
| Scopus | 2000-2020 | 3 Aug 2020 | 37 | 10 |  |
| Web of Science | 2000-2020 | 4 July | 181 | 16 |  |
| **Total** |  |  | **1327** | **137** |  |
| Removing duplicates |  |  |  | 81 |  |

## **EBSCO (CINAHL, EconLit, Health Source: Nursing/Academic Edition, Humanities Source, MEDLINE, APA PsycArticles, APA PsycInfo, Social Work Abstracts)*

**Appendix 2. Data abstraction form**

**Reviewer: _______________________________ Date of data extraction:** ____________

**Bibliographic details of study (author.Year.Title.Journal/vol,no.pg)**

**Purpose of study:** ________________________________________________________________________

________________________________________________________________________

**Study design (methods/methodology):** ________________________________________________________________________

**Participants______________________________________________________________**

**Country of Study: _______________________________________________**

**Data Analysis______________________________**

**Health financing reform process examined _________________________________________________________**

**Outcomes of the health financing reform _____________________________**

**Role of the SDGs declaration in the reform**

**________________________________________________________________________**

**________________________________________________________________________**

**Comments_________________________________________________________________**

**Complete. Yes______ No_____**

| Findings | Illustration form publication (page number) |
| --- | --- |
|  |  |
|  |  |
|  |  |
|  |  |
|  |  |

Extraction of findings complete. Yes______ No_____________

**Appendix 3. Thematic framework analysis format for summarizing changes in health financing occasioned by SDGs declaration**

| **Study** | **How SDGs has been used to/or influenced financing reforms (emerging themes)** | **Aspects of the health financing reformed or being reformed (health financing sub-functions* organization and management)** | **Key issues or lessons and impact (quality of care, financial protection, equity)** |
| --- | --- | --- | --- |
|  |  |  |  |
|  |  |  |  |
|  |  |  |  |
|  |  |  |  |

*revenue collection, revenue pooling, benefits design and service purchasing

**Appendix 4. Excluded studies and reasons for exclusion**

| **Code** | **Reason for Exclusion of the article** | **Number of articles** |
| --- | --- | --- |
| 1 | Discuses health financing reforms not in the context of SDGs declaration | 28 |
| 2 | Not a health financing study but discusses other UHC dimension like improving services coverage | 29 |
| 3 | Study on trends in health financing indicators with no discussion on how it has been affected by SDGs declaration | 9 |
| 4 | Studies on SDGs not related to health financing reforms | 5 |
|  | ***Total*** | ***71*** |

| **No** | **Article** | **Coded reason for exclusion** |
| --- | --- | --- |
| 1 | Advancing universal health coverage in South Asian cities: A framework | 1 |
| 2 | The Dynamics of Catastrophic and Impoverishing Health Spending  in Indonesia: How Well Does the Indonesian Health Care Financing System Perform? | 3 |
| 3 | Health status and health systems financing in the MENA region: roadmap to universal health coverage | 3 |
| 4 | Universal Health Coverage: Assessing  Service Coverage and Financial Protection for All | 1 |
| 5 | Financial sustainability versus access and quality in a challenged health system: an examination of the capitation policy debate in Ghana | 1 |
| 6 | Measuring progress towards Sustainable Development Goal 3.8 on universal health coverage in Kenya | 3 |
| 7 | Assessing Fiscal Space for Health in the SDG Era: A Different Story | 1 |
| 8 | Understanding the implications of the Sustainable Development Goals for health policy and systems research: results of a research priority setting exercise | 4 |
| 9 | Next steps towards universal health coverage call for global leadership | 2 |
| 10 | Health financing strategies to reduce out- of-pocket burden in India: a comparative study of three states | 2 |
| 11 | Myriad of Health Care Financing Reforms in Zambia: Have the Poor Benefited? | 2 |
| 12 | Health Financing Reforms for Moving towards Universal Health Coverage in the Western Pacific Region | 1 |
| 13 | The political economy of health financing reform in Malaysia | 1 |
| 14 | Policy analysis of the Iranian Health Transformation Plan in primary healthcare | 3 |
| 15 | Investigating causality on the path to Universal Health Coverage | 2 |
| 16 | Economou C, Kaitelidou D, Karanikolos M, Maresso A. Greece: Health system review. *Health Systems in Transition*, 2017; 19(5):1–192. | 3 |
| 17 | Implementation Research to Strengthen Health Care Financing Reforms Toward Universal Health Coverage in Indonesia: A Mixed-Methods Approach to Real-World Monitoring | 1 |
| 18 | Achieving universal health coverage in South Africa through a district health system approach: conflicting ideologies of health care provision | 2 |
| 19 | The Rise and Fall of “Universal Health Coverage” as a Goal of International Health Politics, 1925–1952 | 2 |
| 20 | Health care reforms and changes in health expenditure in a period of financial crisis | 3 |
| 21 | Development of village doctors in China:  financial compensation and health system  support | 1 |
| 22 | The politics of the basic benefit package health reforms in Tajikistan | 2 |
| 23 | Impacts of Health Reform Plan in Iran on Health Payments  Distributions and Catastrophic Expenditure | 1 |
| 24 | “We are called the et cetera”: experiences  of the poor with health financing reforms  that target them in Kenya | 1 |
| 25 | Catastrophic healthcare expenditure and  poverty related to out-of-pocket payments  for healthcare in Bangladesh—an estimation  of financial risk protection of universal health  coverage | 3 |
| 26 | Ethical Consideration of National Health Insurance Reform for Universal Health Coverage in the Republic of Korea | 1 |
| 27 | Rethinking Global Health Governance in a Changing World Order for Achieving Sustainable Development: The Role and Potential of the ‘Rising Powers’ | 4 |
| 28 | Forty years of reform and opening up: China’s progress toward a sustainable path | 2 |
| 29 | The Economic Transition of Health in Africa: A Call for Progressive Pragmatism to Shape the Future of Health Financing | 1 |
| 30 | Remembering Alma-Ata: challenges and innovations in primary health care in a middle-income city in Latin America | 2 |
| 31 | Pacific island health inequities forecast to grow unless profound changes are made to health systems in the region | 2 |
| 32 | Financing intersectoral action for health: a systematic review of co-financing models | 1 |
| 33 | What can we learn from China’s health system reform? | 2 |
| 34 | Who pays for healthcare in Bangladesh? An analysis of progressivity in health systems financing | 3 |
| 35 | Decomposing inequality in financial protection situation in Iran after implementing the health reform plan: What does the evidence show based on national survey of households' budget? | 3 |
| 36 | The New Era of Health Goals: Universal Health Coverage as a Pathway to the Sustainable Development Goals | 2 |
| 37 | Reforms of the health care financing system in Poland introduced between 2016 and 2018 | 1 |
| 38 | Fiscal space for sustainable financing of health systems and universal health | 2 |
| 39 | Legislating for universal access to medicines: a  rights-based cross-national comparison of UHC  laws in 16 countries | 2 |
| 40 | Primary Health Care That Works: The Costa Rican Experience | 2 |
| 41 | HEALTH FINANCING POLICY REFORM TRENDS: THE CASE OF LATVIA | 1 |
| 42 | Progress Toward Universal Health Coverage  A Comparative Analysis in 5 South Asian Countries | 2 |
| 43 | Challenges and opportunities towards the  Open Accessroad of universal health coverage (UHC) in Nepal: a systematic review | 2 |
| 44 | Looking at the bigger picture: how the wider health financing context affects the implementation of the Tanzanian Community Health Funds | 1 |
| 45 | Health insurance reforms in Singapore and Hong Kong: How the two ageing asian tigers respond to health financing challenges? | 1 |
| 46 | How much should Hong Kong spend on its healthcare? | 1 |
| 47 | 10 years of China’s comprehensive health reform: a systems perspective | 2 |
| 48 | Why strengthening primary health care is  essential to achieving universal health coverage | 2 |
| 49 | Sub-national health care financing reforms in Indonesia | 1 |
| 50 | Russo G, Bloom G, McCoy D. Universal health coverage, economic slowdown and system resilience: Africa’s policy dilemma. BMJ Global Health. 2017;2(3):e000400. | 2 |
| 51 | Lebanon's essential health care benefit package: A gateway for universal health coverage | 2 |
| 52 | Healthcare financing in South-East Asia: Does fiscal capacity matter? | 1 |
| 53 | Social health insurance development in Mongolia: Opportunities and challenges in moving towards Universal Health Coverage | 1 |
| 54 | Moving Towards Universal Health Coverage in Haiti | 1 |
| 55 | Transforming health systems financing in Lower Mekong: making sure the poor are not left behind | 1 |
| 56 | High-quality health systems in the Sustainable Development Goals era: time for a revolution | 4 |
| 57 | Universal health coverage and the health Sustainable Development Goal: achievements and challenges for Sri Lanka Amala de Silva1, Thushara Ranasinghe2, Palitha Abeykoon3 | 2 |
| 58 | Perera S, Nieveras O, de Silva P, Wijesundara C, Pendse R. Accelerating reforms of primary health care towards universal health coverage in Sri Lanka. WHO South-East Asia J Public Health. 2019;8(1):21–5. | 2 |
| 59 | Assan A, Takian A, Aikins M, et al. Challenges to achieving universal health coverage through community-based health planning and services delivery approach: a qualitative study in Ghana. BMJ Open. 2019;9:e024845. https://doi.org/10.1136/bmjopen-2018-024845. | 2 |
| 60 | Pisani E, Olivier Kok M, Nugroho K. Indonesia’s road to universal health coverage: a political journey. Health Policy Plan. 2017;32(2):267–76. | 2 |
| 61 | Lagomarsino G, Garabrant A, Adyas A, Muga R, Otoo N. Moving towards universal health coverage: health insurance reforms in nine developing countries in Africa and Asia. Lancet. 2012;380(9845):933–43. | 1 |
| 62 | Maher A, Bahadori M, Ravangard R. The integration of health insurance funds as a reform approach in Iran. Shiraz E-Medical J. 2017;18(2). https://doi.org/10.17795/semj45600 | 1 |
| 63 | Tangcharoensathien V, Witthayapipopsakul W, Panichkriangkrai W, Patcharanarumol W, Mills A. Health systems development in Thailand: a solid platform for successful implementation of universal health coverage. Lancet. 2018; 391(10126):1205-1223. https://doi.org/10.1016/S0140-6736(18)30198-3 | 2 |
| 64 | Akda R. Lessons from health transformation in Turkey: leadership and challenges. Health Syst Reform. 2015;1(1):3-8. https://doi.org/10.1080/23288604.2014.956017 | 2 |
| 65 | Kuhonta EM. The Politics of Health Care Reform in Thailand. Towards Universal Health Care in Emerging Economies. Springer. 2017. [Last accessed on 2017 Dec 13]. Available from https://www.link.springer.com/ chapter/10.1057/978-1-137-53377-7_4 . | 2 |
| 66 | Kuhonta EM. The Politics of Health Care Reform in Thailand. Towards Universal Health Care in Emerging Economies. Springer. 2017. [Last accessed on 2017 Dec 13]. Available from https://www.link.springer.com/ chapter/10.1057/978-1-137-53377-7_4 . | 2 |
| 67 | Fahim, Shah Mohammad. Financing health care in Bangladesh: Policy responses and challenges towards achieving universal health coverage | 1 |
| 68 | Financing the SDGs: mobilizing and using domestic resources for health and human capital | 4 |
| 69 | Towards a coherent global framework for health financing: recommendations and recent developments | 1 |
| 70 | Current situation and progress toward the 2030 health-related Sustainable Development Goals in China: A systematic analysis (Not health financing related to SDGs | 4 |
| 71 | Pokharel R, Silwal PR. Social health insurance in Nepal: a health system departure toward the universal health coverage. Int J Health Plann Manag. 2018. https://doi.org/10.1002/hpm.2530 | 1 |
